# Analyzing the shift to the far right: the Austrian case

**DOI:** 10.1057/s41311-023-00443-x

**Published:** 2023-02-27

**Authors:** Ruth Wodak

**Affiliations:** 1 Department of Linguistics and English Language, Lancaster University, County South, LA1 4YL Lancaster, UK; 2 Institut für Sprachwissenschaft, Vienna University, Sensengasse 3a, 1090 Vienna, Austria

**Keywords:** Austrian Peoples Party (ÖVP), Propaganda, Shameless normalisation, Message control, Demagogy, Press freedom

## Normalizing authoritarianism

Since the beginning of the twenty-first century, authoritarianism and ‘illiberal democracies’ have become ever more acceptable and indeed normalized in the European Union and beyond (Wodak 2021). The checks and balances, democratic institutions, are being systematically undermined, including media, justice, academia, and education in Hungary, Poland, and Turkey 1. Although far-right populist parties often call for more ‘direct democracy’, new legislation is quickly implemented without accounting for transparency, expert opinions, minority rights and opposition, and so forth. In fact, Luke Cooper (2021, 7) argues that “once authoritarian forces gain a foothold, they are hard to dislodge. Financial resources can be raised from supporters and access to the mainstream media assured (Wodak 2022). Even when authoritarians are outside of government, they can assert a strong hold on political culture as an opposition bloc and threat to the status quo.” Indeed, illiberal practices have become normalized, employed by conservative parties and their autocratically minded leaders. Accordingly, Jan-Werner Muller maintains that the main aim is accessing power, frequently without pursuing distinct ideologies and agendas, in other words: remaining in power by whatever means necessary.^2^ Poignantly, he labels this kind of regime as ‘conservativism without qualities’ [*Konservatismus ohne Eigenschaften*], a reference to Robert Musil’s seminal novel from the 1930s about ‘the typical Austrian’—*The Man without Qualities* [*Der Mann ohne Eigenschaften*].

In this essay, I focus on one of the many ways propagandistic tools are employed to control the relevant agenda and information being disseminated by both traditional media (broadsheets, tabloids, public TV, and radio) and online (Facebook, Twitter, Instagram, YouTube, blogs, and so forth): in other words, on *message control*, as part of the shift to the (far) right, to authoritarianism. The concept of ‘message control’ emerged from the specific propaganda tool developed by the former Austrian Chancellor Sebastian Kurz and his advisors, and implies launching and thus controlling information via weekly press conferences, briefings, personal conversations, backroom debates (*Hintergrundgespräche*), and text messages, and to financially subsidize only those media that reported favorably about the activities of Kurz’s government. A new media logic based on favoritism, nepotism, and clientelism was established and normalized (Pilz 2021), like Hungarian Prime Minister’s Viktor Orbán’s attack on all independent media in Hungary (Wodak 2021, 227*ff.*). This stands in contrast to Trumpism, which delegitimized all investigative journalism without explicitly attempting to control it (Wodak 2022).^3^

Before analyzing massage control in more detail, I will first point to the violation of norms and values, the *shameless normalization* of previously tabooed agendas, and the shifting of the boundaries of the unsayable as a *strategic facilitator and predictable consequence* of message control. Moreover, the limitations of message control will be discussed in times of social media and multiple public spaces. Obviously, an effective top-down control of media proves impossible in liberal democracies.

## Provocation, Scandalization, and ‘Coarse Civility’

In 1970, Herbert Marcuse wrote in his preface to the second edition of *Prophets of Deceit*, Leo Löwenthal and Norbert Guterman’s seminal study on the main characteristics, performance, and effects of virulent demagogy originally published in the aftermath of World War Two:

The demarcation line between the outsider agitator and the legitimate politician, between the extreme right and the center [is] being blurred (if not obliterated). Today, we recognize the essential features of the agitator as those of the political Establishment. (Marcuse 2021 [1969], xlii).

This echoes Theodor W. Adorno’s statement (1969, 364–365) that

“[t]he calculated influence of agitators on the ‘lunatic fringe’ is by no means the only and probably not even the most important objective factor promoting a fascistically inclined mentality among the masses”.

Indeed, Adorno claims (ibid.) that the opinions of such demagogues “occur in considerable measure in the utterances of so-called ‘respectable’ people, only not as succinctly and aggressively formulated”. Both Marcuse and Adorno were referring to the US-politics and media during the Vietnam War, thus to phenomena pertaining to postwar democratic systems and not to the totalitarian regimes and their leaders as originally analyzed by Löwenthal and Guterman (2021/1949). Nevertheless, these poignant insights remain relevant to scholars who are investigating the ‘(shameless) normalization’, ‘recontextualization’, ‘contagion’, and ‘mainstreaming’ of far-right and extreme-right tropes, arguments, topics, and agendas in liberal democracies (e.g., Cooper 2021; Fielitz and Marcks 2019; Fuchs 2018; Krzyżanowski 2020; Mondon & Winter 2021; Rheindorf and Wodak 2019; Wodak 2019, 2021).

In illiberal democracies, most breaches of the constitutional order, such as freedom of opinion, assembly, the press, and independence of the legal system (as are occurring especially in Poland and Hungary), are usually not announced explicitly. Indeed, as (Fournier 2019, 366) maintains, “[t]he populist rhetoric manipulates the rule of law and the majoritarian pillars of constitutional democracy by convincing a fictional majority that constitutional democracy gives rise to a tyranny of minorities”. Accordingly, the far-right (populist) media strategy functions, I claim, as a *catalyst*, an instrument of mobilization, distraction, and subsequently, of normalization (Wodak 2021, 56).

Wilhelm Heitmeyer’s theory of ‘coarse civility’ [*rohe Bürgerlichkeit’*] (2018) points to the important contribution of conservative elites and the media in shifting the boundaries of normalcy. According to Heitmeyer, such elites can, on the one hand, repeatedly re-establish and strengthen ‘fundamental values’ even in times of great uncertainty; on the other hand, they can contribute to the relaxation of these very fundamental values. This is achieved by, among other things, “the placement of terms or catchy formulas” (ibid., 294) that recontextualize formulations. Heitmeyer demonstrates the emergence of new meanings and interpretations, especially by important actors in public life (‘transmission actors’) (ibid., 295). Krzyżanowski and Ledin (2017), on the other hand, analyze the blurring of boundaries between civility and uncivility, and illustrate that much ambivalence accompanies so-called *discursive shifts*, i.e., a borderline discourse, “which verges on civility and uncivility, in context-dependent ways” (ibid.).

My own definition of *shameless normalization* refers to ‘blunt or shameless discursive and material practices’ (Wodak 2021, 6). Obviously, the far-right populists’ agendas (and related rhetoric) have already reached the political mainstream. Hence, everybody is confronted with widespread normalization of far-right policies, of formerly tabooed topics, wordings, and impolite^4^ behavior (i.e., ‘bad manners’). Traditional norms and rules of political culture, of negotiation and deliberation, are violated by continuous provocations, disseminated via the media, supported by mainstream conservatives, and thus—shamelessly—normalized.

Importantly, Jörg Flecker’s empirical study (2020) illustrates that views of society and political orientations at the center or mainstream are not straightforward, but often ambivalent, even partly contradictory. Flecker’s arguments relate well to Cooper’s claims about ‘authoritarian protectionism’ (2021, *13ff.*). Flecker emphasizes two salient developments since the 1990s (which became even more relevant following the financial crisis in 2008) that prove dangerous to liberal democracies: first, a tendency to endorse nativist, identitarian thinking; and second, orientation toward authoritarianism. Normalization of far-right populist ideologies lead to ever stronger ethno-nationalist views: belonging to a collective is seen as linked to biological heritage and ancestry. Shifts in the messages, framing, and discourse can have a substantial impact—including, but not necessarily, on normalizing far-right positions (ibid.). In the extreme form of cultural racism, this goes hand in hand with the debasement of the other as inferior. Flecker maintains that such cultural racism has been empirically found among those who, for instance, believe that specific groups of migrants are not suited to wage labor. This implies that: ‘Whoever doesn’t have their roots here shouldn’t stay’. Ever more explicit authoritarian attitudes can be observed, expressed toward migrants as well as long-time unemployed people, homeless people, and people who receive social benefits. Slogans that call for ‘cracking down’ or ‘getting tough’ to ‘move’ people to take on jobs resonate widely (ibid.). Accordingly, such attitudes reflect the impact of neoliberal policies as well as rising inequality, both of which support the shameless normalization of the far right, widely disseminated by traditional and social media, and frequently drawing on traditional antisemitic stereotypes that imply some form of Jewish world conspiracy, e.g., on anti-Sorosism (e.g., Wodak 2021, 139–140; Cooper 2021, 34).

## ‘Message Control’ and ‘Tabloid-democracy’

In contrast to Donald Trump’s strategy as an agitator who created his own ‘fake’ news and circumvented and delegitimized investigative journalism, Sebastian Kurz, who served as Austria’s Chancellor until October 9, 2021, was a master of image-building who attempted to *control* the media. In the four years since he first took over leadership of the national-conservative Austrian People’s Party (ÖVP) at just 30 years of age in 2017, he raised it to new electoral heights by combining hardline immigration rhetoric with personality-driven politics and calls for a ‘new style’ of governing.^5^ The analysis presented here follows Douglas Walton’s taxonomy of argumentative schemes of ‘doing propaganda’ (1997),^6^ where he distinguishes ten essential characteristics. In this analysis, due to reasons of space, I can only point to some important dimensions (Table 1).

**Table 1:** Relevant propagandistic strategies for message control

*Message content: argument + persuasion***:** Believe something you did not believe before
*Dialogic structure***:** Argumentum ad populum
*Goal-directed structure***:** Get the respondent to carry out/support a particular action
Legitimation + indifference to logical reasoning: *Appeals to authorities, intuition, and emotions*
*Orchestration*: Manipulation of all accessible and different media channels to produce a cumulative message

### Controlling message content

Message control implies a relentless internal focus on ensuring that a certain narrative about, and perception of, the government is disseminated in media and public life.^7^ For example, Sebastian Kurz launched his 2017 national election campaign with a so-called *big lie*.^8^ He claimed to have closed the so-called Balkan route for refugees traveling to Europe; he argued that he had prevented even more refugees entering the European Union. At every possible occasion, this lie was repeated, and he was widely acclaimed as the savior of the Occident. This argument is fallacious since—according to facts—Angela Merkel’s initiated 2016 deal between Europe and Turkey (more or less) closed the Balkan route when Turkey promised to provide protection to refugees arriving from Syria and Iraq; in return, Turkey received 6 billion Euro (until 2018) to support these refugees.^9^ Thus, refugees did not have to flee to Austria, Germany, or Sweden, they had to remain in Turkey.

Kurz also had a habit of calling up journalists directly or having aides reach out on his behalf when he felt their stories were too critical about the government’s policies. Once Kurz became Chancellor, he hired 80 PR consultants under the supervision of his personal advisor, Gerhard Fleischmann, who were responsible for implementing his strategic agenda. The same is true for the Ministry of Interior Affairs which also hired 80 PR consultants. In this way, *unilateral narratives* were disseminated, without critical discussion or counter-arguments.^10^ The ORF, the state broadcaster, one of whose news divisions is sometimes critical of the government, remains on stable financial footing, thanks to its financing mix of mandatory license fees from viewers and advertising. But journalists in the ORF report that the broadcaster faced continuous interventions from Kurz’s government in news coverage. Indeed, the election of a new ORF director, which the ÖVP could directly influence through the board of governors, resulted in the installation of a political crony in August 2021.^11^

Furthermore, as new evidence brought to light in October 2021 illustrates, Kurz and his followers allegedly used taxpayer money to pay for manipulated polls, thus exaggerating their support. The publication of text exchanges (WhatsApp) from within Kurz’s loyal inner circle has made explicit the lengths to which the former Chancellor was willing to go to steer the media.^12^ One of the salient text messages “Who pays, gives the orders” (“*wer zahlt, schafft an*”)^13^ describes the *leitmotif* of the corrupt dealings with the media: Kurz and his team paid; and the respective media published the strategically placed positive and uncritical reports. Endorsed with a huge budget and communication staff, Kurz’s media operation was indeed larger than many Austrian newsrooms (Schultheis 2021). The government coalition which Kurz led until October 9, 2021, which includes his party, the ÖVP, and the Greens, earmarked €210 million for media spending until 2024. As Karnitschnig (2021) rightly summarized after Kurz’s resignation on October 9, 2021:

Whether Kurz will succeed in rescuing his reputation is another question. He seemed particularly embarrassed by text exchanges published in court filings between him and close aides that belie the public image he has cultivated over the years as a selfless public servant. In the texts, Kurz comes across as a cutthroat political operator willing to do whatever it takes to win power.^14^

### Autocratic pressure

Related to *content control* are the many ways in which Kurz and his party sought to subvert or undermine democratic institutions whenever this would serve their political interests. The undermining of checks and balances follows an obvious striving for power, at the same time neglecting previous constitutive principles of the party which had always defined itself as ‘Christian social’ and thus abiding by Christian humanitarian values.

Accordingly, since 2020, Kurz and his followers have repeatedly attacked Austria’s judiciary as politically motivated, specifically by ‘left-wing networks’ and ‘left-wing conspiracies’.^15^ However, no evidence exists for such networks. This accusation was repeated at every possible occasion so that—unsurprisingly—many people started to challenge the independence of the judiciary. Moreover, after several policy measures implemented during the COVID-19 pandemic were judged illegal by the Supreme Court, Kurz derogatorily maintained that supreme judges’ decisions were only “legal quibbles” and not to be taken seriously.^16^

The pressure on media reporting also has huge financial implications. Subsidizing friendly media and punishing critical media blatantly instrumentalizes the volatile situation of (Austrian) media. Most newspapers, apart from the tabloids, are experiencing massive economic problems due to global, national, and glocal transformations of traditional media audiences. Hence, if a newspaper decides to remain critical, it might end up depending solely on its outreach and sales, and not receive any subsidies by the state. In this way, critical media are being intentionally *starved*. In the past, most of the subsidies was spent on advertising, a practice - critics contend - that should be seen as masked support for the country’s powerful tabloids, which endorsed Kurz and have received most of the cash; critical media received much less, if they received anything at all. In 2020, for example, the coalition spent €47 million on such advertising, or triple what the previous government did.^17^

Against this background, the concept of ‘Boulevard-democracy’ was coined by political scientist Fritz Plasser^18^ to describe how the placement of advertisements leads to a toxic symbiosis of government and tabloids. A paradox obviously, because the fourth estate should take a critical look at, and challenge, those in power. According to investigative journalist Eva Linsinger (2021, 30ff.), the opposite is common in Austria. The respective rulers are adored and presented in exaggerated positive terms. For example, with headlines like "Abroad they love the Wonder Boy Kurz" (*OE* 24, 18 January 2018^19^), related to some German media and politicians who wished for a similar German “wonder boy”.

Quite like Viktor Orbán’s regime (e.g., Cooper 2021, 102), Kurz and his followers assisted wealthy supporters when buying (parts of) relevant media, especially tabloids. This strategy necessarily guarantees friendly reporting: For example, René Benko, one of the richest men in Austria, bought a 49 percent stake in the WAZ Holding GmbH which owns 50 percent of the tabloid with the largest outreach, *Neue Kronen Zeitung*, and almot 50 percent of *Kurier*, the most government-friendly newspaper. At the same time, he took the critical online newspaper *ZackZack* to court, accusing it of libel and demanding €1 million as compensation—a sum which would have necessarily destroyed *ZackZack*; but *ZackZack* was able to win the court case. 20

### Orchestration and legitimation of political agenda

To attract voters from the extreme-right Freedom Party (FPÖ), Kurz strategically blurred the boundaries between far-right populist rhetoric and traditional conservative values, thus normalizing far-right policies on immigration and human rights and increasing the polarized political environment. This presents a prime example of coarse civility and of Walton’s strategies of *orchestration and manipulation* of message content. Instead of discussing and providing solutions for major sociopolitical problems such as globally rising inequality and youth unemployment, or the consequences of climate change for migration politics, refugees and migrants continue to serve as *the* scapegoat for all woes.

National-conservative parties present themselves as the ‘soft, politically correct alternative’ to far-right populism and indeed, demagoguery. If, for example, Sebastian Kurz in his role as Chancellor justified, as he did in 2018, cutting support for the poorest in society by alleging that in the families of unemployed persons it was “only the children who get up in the morning, to go to school”^21^ or denounced the saving of lives in the Mediterranean as “NGO madness”,^22^ then such utterances correspond to the shameless normalization of far-right slogans and arguments. Here, the connections and, indeed, overlaps of policies between neoliberal, neoconservative, and far-right ideologies are particularly evident (Krenn 2020,23). These utterances were widely disseminated via message control and resonated with large parts of the Austrian public.

Another propagandistic strategy consists of *inclusion of some and exclusion of others* from important news. Thus, Kurz frequently invited selected journalists to so-called backroom discussions [*Hintergrundgespräche*] to inform them of the ÖVP’s preferred narrative, thus intentionally distracting journalists’ attention from other agendas (the so-called ‘dead cat strategy’^24^). Critical journalists were literally not allowed to participate and enter the room. Friendly journalists widely disseminated intentionally constructed “mind-closing narratives” (Grabbe and Lehne 2017). Moreover, access to information was being severely restricted: government employees were frequently forbidden to speak with the press. Such strategies come close to so-called managed democracies and their press policies (Wodak 2019).

## Normalization of control?

The shameless normalization of far-right agendas leads to a step-by-step implementation of authoritarianism in liberal democracies. Investigative journalism has come under strong pressure—via exclusion from access to information; via delegitimization of their work; via financial pressure and even, as recent developments in Hungary, Turkey, and Poland illustrate, via closing down independent media channels and imprisonment—or indeed assassination (for example, in Malta, Slovakia, and Russia) of journalists.^25^ In view of the above, Scheppele (2021, 547–548) rightly argues:

Democracies are not just failing for cultural or economic or political reasons. Some constitutional democracies are being deliberately hijacked by a set of legally clever autocrats, who use constitutionalism and democracy to destroy both [...] Because these autocrats push their illiberal measures with electoral backing and use constitutional or legal methods to accomplish their aims, they can hide their autocratic designs in the pluralism of legitimate legal forms.

Critically challenging what is otherwise taken for granted and essentialized should therefore be regarded as a first step to protecting press freedom and independent scholarship.

## Footnotes

1 See the 2021 annual report of ROG (*Reporter ohne Grenzen*) concerning Hungary’s violated press freedom (http://netzpolitik.org/2021/feinde-der-pressefreiheit-reporter-ohne-grenzen-setzt-orban-auf-die-schwarze-liste/; accessed September 15, 2021). The BBC has also—not surprisingly—encountered interventions from then prime minister Boris Johnson and the Tory Party (e.g., https://www.theguardian.com/commentisfree/2021/jul/20/the-bbc-faces-major-challenges-from-the-government-to-its-independence).

2 https://www.derstandard.at/story/2000130433218/konservatismus-ohne-eigenschaften (accessed November 1, 2021).

3 See Wodak (2022) where Trumpism and Trump’s media logic are contrasted in much detail to Kurz’ ‘message control’; there, a range of empirical examples is provided.

4 Here, I refer to Jonathan Culpeper’s definition of ‘impoliteness’ (which includes hate speech) as “negative attitude towards specific behaviours occurring in specific contexts. It is sustained by expectations, desires and/or beliefs about social organisation, including how one person’s or a group’s identities are mediated by others in interaction” (Wodak et al. 2020, 2).

5 https://www.politico.eu/article/house-of-sebastian-kurz/ (accessed September 10, 2021).

6 See Wodak (2022) for a detailed discussion of Walton’s model.

7 See http://kontrast.at/julia-Herr-sebastian-kurz-median/, where MP Julia Herr of the Social Democratic Party describes relevant stages of ‘message control’ in detail.

8 https://kurier.at/politik/inland/wahl/faktenchek-wer-hat-die-balkanroute-geschlossen/274.540.009 (accessed February 3, 2021).

9 https://www.bundesregierung.de/breg-de/suche/faq-eu-tuerkei-erklaerung-1728136 (accessed November 1, 2021).

10 https://www.politico.eu/article/the-house-of-sebastian-kurz-scandal-explained-austria-chancellor-perjury-allegations/; https://www.politico.eu/article/house-of-sebastian-kurz/ (both accessed October 15, 2021).

11 https://orf.at/stories/3224290/ (accessed October 17, 2021).

12 https://exxpress.at/welches-weib-waere-gut-die-chats-die-uns-vorenthalten-wurden/ (accessed October 19, 2021).

13 https://www.nachrichten.at/nachrichten/fotogalerien/politik/wer-zahlt-schafft-an-die-zitate-der-woche;cme219268,2553305 (accessed 10 November 2021).

14 https://www.politico.eu/article/austrias-sebastian-kurz-resigns/ (accessed October 15, 2021). Matthew Karnitschnig from the online newspaper *Politico* is probably the most knowledgeable foreign correspondent reporting about Kurz’s corruption scandals and Kurz’s downfall for many months in 2021.

15 https://www.nachrichten.at/politik/innenpolitik/justiz-oevp-ortet-linke-zellen-in-der-wksta;art385,3470046 (accessed October 15, 2021).

16 https://www.sueddeutsche.de/meinung/oesterreich-kurz-verfassungskrise-grundrechte-1.5280641 (accessed November 1, 2021); https://www.ots.at/topthema/vfgh-zu-coronamassnahmen (accessed November 1, 2021); https://www.zeit.de/zustimmung?url=https%3A%2F%2Fwww.zeit.de%2Fpolitik%2Fausland%2F2021-05%2Fsebastian-kurz-falschaussage-oesterreich-ibiza-affaere-untersuchungsausschuss%2Fseite-2 (accessed November 1, 2021).

17 https://www.tortoisemedia.com/2021/10/22/the-fall-of-austrias-sebastian-kurz-has-lessons-for-every-democracy/ (accessed October 29, 2021).

18 https://www.zeit.de/kultur/2019-05/medien-oesterreich-kronenzeitung-boulevarddemokratie-heinz-christian-strache (accessed October 29, 2021).

19 https://www.oe24.at/oesterreich/politik/wolfgangfellner/im-ausland-lieben-sie-den-wonder-boy-kurz/317931272 (accessed October 15, 2021). By the way, the tabloid *Österreich* and TV station *OE 24* belong to Wolfgang Fellner who allegedly was most subsidized by the ÖVP and published the manipulated surveys mentioned above.

20 https://www.krone.at/2484607; https://zackzack.at/2021/08/13/gewinnen-wir-gemeinsam-gegen-benko/ (both accessed October 15, 2021).

21 See https://orf.at/stories/3107219/ (accessed January 15, 2020).

22 See www.derstandard.at/story/2000054765748/kurz-kritisiert-rettungsaktionen-von-ngos-im-mittelmeer-scharf (accessed January 15, 2020).

23 See the blog by Manfred Krenn (2020) ‘Rechtsextremismus in der Normalität—Alarmismus oder notwendiger Weckruf?’ (https://awblog.at/rechtsextremismus-in-der-normalitaet/; accessed January 15, 2020).

24 See www.politics.co.uk/blogs/2013/11/24/the-dead-cat-strategy-how-the-tories-hope-to-winthe- next-election/ (accessed November 3, 2021).

25 http://www.centeronnationalsecurity.org/vital-interests-issue-22-kim-lane-scheppele (accessed November 2, 2021).
